# Myocarditis Prognostic Score: A New Risk Assessment Tool

**DOI:** 10.3390/jcdd13060223

**Published:** 2026-05-23

**Authors:** Daniela Di Lisi, Cristina Madaudo, Francesca Macaione, Francesca Castro, Francesco Bongiorno, Francesco Stabile, Andrea Micarelli, Alfredo Ruggero Galassi, Giuseppina Novo

**Affiliations:** 1 Division of Cardiology, University Hospital Paolo Giaccone, 90127 Palermo, Italy; 2 Department of Health Promotion, Mother and Child Care, Internal Medicine and Medical Specialties (PROMISE) “G. D’Alessandro”, University of Palermo, 90133 Palermo, Italy

**Keywords:** myocarditis, score, cardiac magnetic resonance, prognosis

## Abstract

Background: Myocarditis is an inflammatory disease of the myocardium with multiple causes and evolutions. The aim of our study was to design a prognostic multiparametric score in patients with myocarditis, to identify those at higher risk of cardiovascular outcomes. Methods: A prospective study was performed enrolling 98 patients with myocarditis: 72 M, 26 F; median age 27 [IQR 20–40]. Patients were divided into two groups: complicated (CM) and uncomplicated myocarditis (UM). Six months after hospital admission, cardiac magnetic resonance (CMR) and cardiological consultation were repeated. Cardiovascular outcomes (death, hospitalization for heart failure, heart transplant, ICD implantation, and heart failure development) were evaluated at 6 months and after 3 years. Results: We found 67 UM and 31 CM. Cardiovascular outcomes were significantly higher in patients with CM. We found a significant correlation between cardiovascular outcomes and reduced LVEF at hospital admission, reduced global longitudinal strain in absolute values, septal late gadolinium enhancement (LGE) at CMR, longer persistence time of increased troponin, LGE extension progression or persistence at 6 months of CMR. A myocarditis prognostic score was developed. A score ≥ 5 showed higher sensitivity (100%) and specificity (87%)—AUC 1, to identify cardiovascular outcomes in patients with myocarditis. A score between 3 and 4 showed high sensitivity but low specificity. A score ≤ 2 was associated with low probability of cardiovascular outcomes. Conclusion: Our study confirms the high probability of cardiovascular outcomes in patients with CM and it suggests a myocarditis prognostic score to identify patients at higher risk of cardiovascular outcomes.

## 1. Introduction

Myocarditis is an inflammatory disease of the myocardium with multiple causes and multiple evolutions [1]. It can resolve spontaneously without leaving a trace or progress towards dilated cardiomyopathy and heart failure (HF) [2,3]. The immune system plays a central role in the pathogenesis of myocarditis. Independent of the cause of acute myocarditis, an abnormal immune response is considered central [4].

Myocarditis can be infectious and non-infectious. Influenza-associated myocarditis generally carries a more favorable prognosis compared with myocarditis secondary to DRESS syndrome [1].

In the recent 2025 ESC guidelines for the management of myocarditis and pericarditis, the term ‘inflammatory myopericardial syndrome’ was used to reflect the possible myocarditis–pericarditis overlap. Particularly, in myopericarditis, pericarditis is predominant; in perimyocarditis, myocarditis is predominant, with newly developed regional or global impairment of left ventricular function in echocardiography or cardiac magnetic resonance (CMR) [5].

In these ESC Guidelines, myocarditis was stratified into low, intermediate and high risk based on LVEF values, extensive LGE on CMR, symptoms, ventricular arrhythmias and A-V block [5].

Several prognostic stratifications exist in patients with myocarditis and numerous gaps remain.

Based on the clinical presentation at admission, myocarditis can be classified into complicated and uncomplicated [6].

Uncomplicated myocarditis is defined by the presence of chest pain, preserved ventricular ejection fraction (LVEF ≥ 50%), no acute HF, no sustained ventricular arrhythmias, and no advanced conduction disturbances. Complicated myocarditis is defined by: LVEF < 50%, acute HF, ventricular tachycardia/ventricular fibrillation, second- or third-degree atrio-ventricular block, and cardiogenic shock. Fulminant myocarditis is characterized by hemodynamic instability [6]. Complicated myocarditis seems to have a worse prognosis [7].

Several studies showed the prognostic role of CMR parameters, echocardiographic/electrocardiographic parameters and laboratory test in patients with myocarditis.

For example, high levels of NT-proBNP have a prognostic role in patients with myocarditis [8].

The prognostic role of troponin is not very clear. Absolute troponin values and their trend are only roughly related with myocarditis severity and prognosis. Previous studies showed that an early rise and steep decline of high sensitivity troponin is generally associated with the resolution or at least attenuation of the inflammatory process and with a good prognosis, while recurrently or persistently abnormal high sensitivity troponin values, even if mildly increased, may suggest relapsing or ongoing myocardial damage, as may happen in patients with myocarditis associated with systemic inflammatory disorders or cardiac sarcoidosis [9].

Recently, a new marker (elevated Neutrophil-to-Lymphocyte Ratio) was associated with worse prognosis in patients with acute myocarditis and other diseases [10,11,12].

Left ventricular global longitudinal strain (LV GLS) assessed using echocardiography or CMR seems to provide an independent prognostic value in patients with myocarditis and preserved LVEF [13,14,15].

In addition, myocarditis can be acute or chronic. Chronic inflammatory cardiomyopathy is a myocarditis with left ventricular dysfunction and/or dilatation and onset of symptoms > 30 days [16]. Meanwhile, acute myocarditis is an inflammation of myocardium with symptoms onset < 30 days. CMR is the non-invasive gold standard in the diagnosis of myocarditis [17].

Instead, endomyocardial biopsy (invasive gold standard) is recommended only in patients with unexplained acute cardiomyopathy complicated by hemodynamic instability, advanced degree atrio-ventricular block or ventricular arrhythmias, and in patients who do not respond to medical therapy within 1 to 2 weeks [17,18,19].

CMR is not only important in the diagnosis of myocarditis, but it is also able to provide important prognostic information, as demonstrated in various studies [20].

Thus, considering the known prognostic factors in myocarditis and the classification of myocarditis into “complicated” and “uncomplicated”, the aim of our study was to design a prognostic multiparametric score (including several factors such as LVEF, LGE side and progression, LV GLS and troponin) to identify patients with myocarditis at higher risk of composite outcomes.

Considering that our study was conducted before the publication of these guidelines, we used the old classification in “complicated” and “uncomplicated” myocarditis proposed by Ammirati et al. [6].

## 2. Methods

### 2.1. Patient Selection

**Figure 1:**
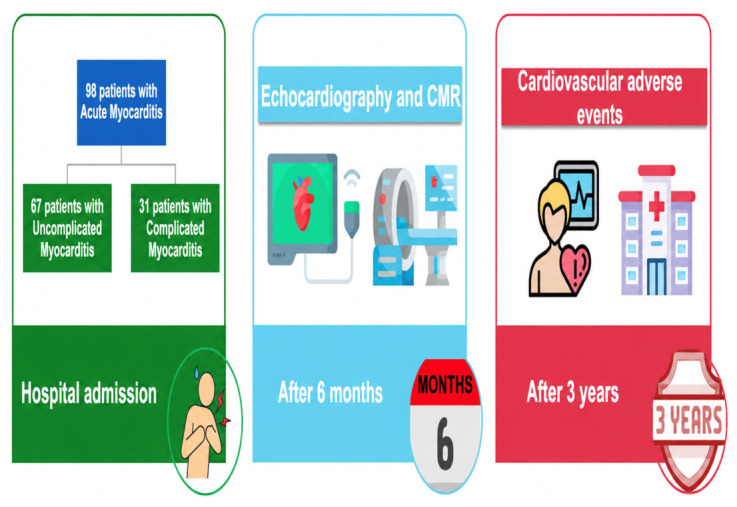
General population and follow-up.

We carried out a prospective study enrolling 98 consecutive patients diagnosed with myocarditis at our institution, between 2016 and 2022 (Figure 1). The study was conducted according to the Declaration of Helsinki and approved by the internal review board. All patients provided informed consent for the conduction of the study.

Inclusion criteria were: age ≥ 18 years and diagnosis of acute myocarditis (myopericarditis or perimyocarditis) based on clinical, electrocardiogram-ECG, echocardiographic and CMR data or diagnosis of acute myocarditis at endomyocardial biopsy.

Exclusion criteria were: concomitant coronary heart diseases, significant valvular heart disease, severe chronic renal failure, and pericarditis without myocarditis.

The diagnosis of acute myocarditis was established according to current ESC guidelines and consensus recommendations [5,6] using clinical presentation (syncope, chest pain, unexplained arrhythmias and dyspnea or fatigue) in addition to ≥1 mandatory positive diagnostic test (by preference CMR) in the absence of significant coronary artery, valvular or congenital heart disease, or other causes. Thus, the diagnosis of definite myocarditis was performed in the presence of symptoms such as a positive endomyocardial biopsy, or in presence of symptoms such as the 2018 Lake Louise criteria at CMR, and cardiac troponin > 99th percentile of the upper reference level with a rise or fall in the level on serial assessment.

ECG, a laboratory test with measurement of high-sensitivity troponin (Ths) and natriuretic peptides (NT-pro BNP), echocardiography with speckle tracking echocardiography analysis (STE) and CMR were performed in all patients.

Troponin was considered positive if >99th percentile of the upper reference level with a rise or fall in the level on serial assessment. Persistently positive troponin was considered if greater than 99th.

### 2.2. Percentile for Several Days

Coronary angiography was performed only in patients aged >40 years old or presence of cardiovascular risk factors (e.g., diabetes, smoking, hypertension, dyslipidemia, and family history of coronary artery disease) and ECG/echocardiogram abnormalities (e.g., ST segment changes and segmental hypokinesia). Coronary tomography was performed in patients with suspected myocarditis and low pre-test probability of coronary artery disease and aged between 30 and 40 years. In younger patients (<30 years old) without cardiovascular risk factors and CMR-positive for myocarditis, neither coronary tomography nor coronary angiography was performed.

Endomyocardial biopsy was performed only in patients with unexplained acute cardiomyopathy complicated by hemodynamic instability, advanced degree atrio-ventricular block, or ventricular arrhythmias, and in patients who did not respond to medical therapy within 1 to 2 weeks.

Patients were divided into two groups: complicated myocarditis and uncomplicated myocarditis (based on the criteria proposed by Ammirati) [6]. Patients with complicated myocarditis had: LVEF < 50%, acute HF, ventricular tachycardia/ventricular fibrillation, second- or third-degree atrio-ventricular block, and cardiogenic shock. Patients with uncomplicated myocarditis had preserved LVEF and none of the above criteria.

### 2.3. Echocardiographic Data

Comprehensive echocardiography was performed at hospital admission and during follow-up in all patients using a GE Vivid E95 ultrasound system prime echocardiography machine and a 4Vc-D (1.4–5.2 MHz) phased-array probe.

All acquisitions and measurements were carried out according to the current guidelines and recommendations of the American Society of Echocardiography and the European Association of Cardiovascular Imaging [21,22]. STE was performed in all patients with good acoustic window and LV GLS was assessed using a semi-automated 2D-strain software package (EchoPacV.202, GE Healthcare, Horten, Norway). LV GLS was obtained after acquiring two-, three- and four-chamber apical views with a frame rate between 60 and 90 frames per second. The regional speckle area of interest was manually adjusted to obtain optimal tracking results. LV GLS was calculated using a 17-segment model. Normal reference values were considered: −21.5 ± 2%, with a lower limit of normal (LLN) of −18% [23].

### 2.4. Cardiac Magnetic Resonance Data

CMR was performed using a 1.5-Tesla scanner (Achieva; Philips Medical System, Best, The Netherlands), featuring an 8-element cardiac phased array receiver surface coil with the patient in the supine position. Functional electrocardiography-gated sequences were acquired during an end-expiratory breath hold. Cardiac size and systolic function were assessed in a stack of contiguous cine of short-axis and horizontal and vertical long-axis slices. Myocardial edema was diagnosed via short tau inversion recovery (STIR) sequences as an area of a myocardial-to-skeletal muscle signal intensity ratio exceeding 1.9. Myocardial fibrosis was evaluated visually using late post-gadolinium enhancement (LGE) sequences. LV myocardium was divided into 17 segments; the location and the number of segments with LGE and the location of the LGE were assessed.

The “septal LGE criterion” was defined as positive if LGE was present in at least one septal segment. In cases of septal LGE, other CMR criteria confirmed the presence of myocarditis and excluded cardiac sarcoidosis. All CMR images were analyzed in consensus by two trained operators evaluating LGE pattern and localization for each patient. Diagnosis of myocarditis was made using the 2018 Lake Louise criteria and based on the presence of at least one T1-based criterion and one T2-based criterion. The T1-based criterion was considered to be positive if an increase in native T1 relaxation times, increase in extracellular volume (ECV), or positive non-ischemic LGE were detected. The T2-based criterion was positive in cases of increased T2 relaxation times or in cases of regional high T2 signal intensities on T2-weighted images or increased global T2 signal intensity ratio [24]. LGE was quantified by counting the number of segments with LGE, after dividing the left ventricle into 17 segments.

### 2.5. Follow-Up and Outcomes

Follow-up was performed 6 months after hospital discharge and after a median period of 3 years.

Cardiological consultation, ECG, echocardiography with STE analysis and CMR were repeated at 6 months. Cardiological consultation with only ECG and echocardiography were also repeated 3 years after. At CMR, the progression and/or the persistence in the number of segments involved by LGE and the regression of edema were evaluated.

Cardiovascular events (sustained and non-sustained ventricular arrhythmias, hospitalization for HF, all-cause death and cardiovascular death, heart transplantation, and development of heart failure) were evaluated during hospitalization, at 6 months and after a median period of 3 years (Figure 1). Recurrence of myocarditis and Implantable Cardioverter Defibrillator (ICD) implantation were also evaluated during follow-up separately, not as major adverse cardiovascular events (MACE). In addition, ICD implantation occurred in patients with other MACE primary outcomes (such as heart failure with reduced ejection fraction or ventricular arrhythmias). Sustained ventricular arrhythmias were diagnosed by performing a 24 h Holter ECG at follow-up.

Unfortunately, the short duration of follow-up was a limitation of the study.

### 2.6. Statistical Analysis

Continuous variables were expressed as mean ± standard deviation, or median (interquartile range) as appropriate; categorical variables were expressed as frequencies (percentages).

For continuous variables, differences between the two groups were analyzed using independent sample Student *t*-tests or Mann–Whitney U test for non-normally distributed variables; for categorical variables, Chi-squared analysis was used.

Pearsons’ coefficient was used to determine the correlation between instrumental parameters and cardiovascular outcomes in patient population.

For the identification of the predictors of cardiovascular outcomes, the different variables between patients with and without cardiovascular events were evaluated. Variance Inflation Factor was used to detect multicollinearity between variables. Continuous variables were examined for linearity with respect to the log-odds of the outcome. Candidate variables were assessed in univariable logistic regression models. A receiver operating characteristic curve (ROC curve) was used to find the capability of LVEF, GLS, days of increase in troponin, septal LGE at CMR, progression or persistence of LGE at 6 months CMR to predict cardiovascular outcomes and to identify the best threshold value.

Cut-offs of these parameters were chosen based on ROC curve analysis.

A multivariate regression model, incorporating, respectively, LVEF, GLS, days of increase in troponin, septal LGE at CMR, and progression or persistence of LGE at 6 months of CMR, was conducted. Results of logistic regression were reported as adjusted odds ratio (OR) and 95% confidence interval (95% CI). The Hosmer–Lemeshow (H-L) goodness of-fit test and C-statistic were used to confirm good calibration and discrimination of the multivariable model.

The scoring system was derived from the β-coefficients of the final multivariate logistic regression model. Each β-coefficient was divided by the smallest coefficient in the model. The resulting values were rounded to the nearest integer to assign weighted points. Variables with stronger associations (higher β-coefficients) were therefore assigned proportionally higher weights. Analyses were performed using GraphPad statistical software (version 8.4.3-471). *p*-values < 0.05 were considered statistically significant.

## 3. Results

### 3.1. Population Description

Clinical, laboratory, electrocardiographic, echocardiographic and CMR characteristics at the time of diagnosis are summarized in Table 1 and Table 2.

Almost all patients were Caucasian, and the vast majority (74%) were male. The median age was 27 [interquartile range (IQR) 20–40] years. Risk factors were present in a minority of patients: Ten patients (10%) had arterial hypertension, 7% diabetes, and 7% dyslipidemia, and 31% of patients were smokers.

The most common symptom complained of at presentation was chest pain (84% of patients). Dyspnea and syncope were present respectively in 17% and 3% of patients, and fever within 30 days in 39% of patients.

Only seven patients had fulminant myocarditis.

Based on the clinical presentation, we found 31 patients with complicated myocarditis (A group) and 67 patients with uncomplicated myocarditis (B group).

Patients with complicated myocarditis were significantly older compared to B group (respectively 38.95 ± 18.33 vs. 29.4 ± 12, *p* value 0.0027); a higher percentage of women was found in A group, although not significantly (28% in A group and 25% in B group, *p* value 0.8). We did not find other differences between the two groups regarding cardiovascular risk factors.

Regarding clinical presentation, chest pain predominated significantly in B group (92% of patients, *p*-value 0.0024); dyspnea (50% of patients, *p*-value < 0.001), syncope (9%, *p*-value 0.04) and fever within 30 days (58%, *p*-value 0.0045) were the most common symptoms in A group. We did not find significant differences in laboratory test (troponin, C-reactive protein—CRP, N-terminal prohormone of brain natriuretic peptide—NT-pro BNP) between the two groups. Troponin and NT-proBNP were higher in patients with “complicated” myocarditis. We only found a longer persistence time of increased troponin in complicated myocarditis (7 ± 3 days) compared to uncomplicated myocarditis (4 ± 2 days, *p*-value < 0.0001).

Endomyocardial biopsy was performed in 64% of patients with complicated myocarditis; coronary angiography was performed in 53% of patients in A group and 27% of patients in B group (*p*-value 0.01). Coronary tomography was performed in 20% of general population and in 48% of patients with complicated myocarditis. Thus, patients with complicated myocarditis who did not undergo coronary angiography performed coronary tomography. These exams did not show obstructive coronary artery diseases. Cardiac magnetic resonance was performed only in 70% of patients with complicated myocarditis, during hospitalization.

Viral infection was the predominant etiology. Considering that endomyocardial biopsy was performed only in 20 patients, only for these patients the true etiology is known: two patients had giant cell myocarditis, one patient had eosinophilic myocarditis, and other patients had viral lymphocytic myocarditis (parvovirus B19, cytomegalovirus). No cases of cardiac sarcoidosis have been diagnosed. Other cases of myocarditis were considered with undetermined etiology.

In addition, we did not find significant differences in ECG abnormalities between the two groups; 10% of patients with complicated myocarditis had third-degree atrio-ventricular. Patients with complicated myocarditis had significantly lower values of LVEF (*p*-value < 0.0001), higher values of left ventricular end-diastolic volume (EDV) at echocardiography and CMR, and lower LV GLS in absolute value (*p*-value < 0.0001) compared to B group.

Pericardial effusion was found in 17% of patients: it was mild in 80% of patients and moderate in 20% of patients.

Regarding medical therapy, a higher percentage of patients with complicated myocarditis assumed beta-blockers and angiotensin-converting enzyme inhibitors (ACE-I) or angiotensin receptor blockers (ARB), compared to patients without complicated myocarditis (Table 1).

Nevertheless, we found low amounts of beta-blockers and ACE-I therapy in the complicated group (53% of patients treated with ACE-I and 43% of patients treated with beta-blockers) because patients with complicated myocarditis did not tolerate these drugs for associated severe arterial hypotension or bradycardia, and atrio-ventricular blocks. Anti-inflammatory therapy was higher in uncomplicated myocarditis (54% of patients) compared to complicated myocarditis (40%) to relieve pain. In fact, chest pain was the most common symptom in patients with uncomplicated myocarditis. Other studies and analysis are needed to assess if anti-inflammatory treatment can influence the outcome.

In addition, 5% of patients with third-degree atrio-ventricular block needed a pacemaker implantation, while for others, it resolved spontaneously.

### 3.2. Description According to the Outcomes

**Figure 2:**
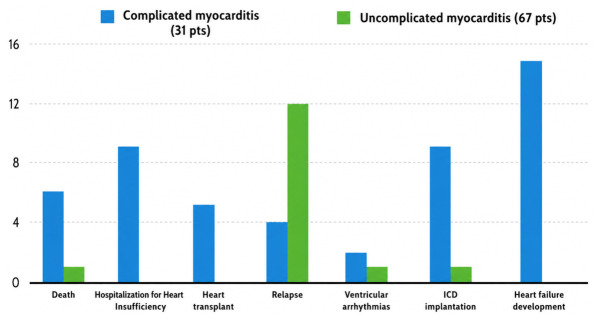
Cardiovascular events in complicated and uncomplicated myocarditis.

Cardiovascular outcomes were reported in 26 patients. We found a higher incidence of composite outcomes in patients with complicated myocarditis compared to patients with uncomplicated myocarditis. A total of 19% of patients of A group had cardiovascular death; no patient in B group had cardiovascular death but only 1% of patients had death from other causes (*p*-value 0.001 between the two groups regarding death from all causes). Patients with complicated myocarditis also had a significantly higher incidence of HF hospitalization (*p*-value 0.01), heart transplant (*p*-value 0.0008), ICD implantation (*p*-value 0.0001) and HF development (*p*-value < 0.0001) compared to patients without complicated myocarditis (Figure 2). ICD implantation was performed in 85% of patients as primary prevention (severe left ventricular dysfunction after optimized medical therapy) and in 15% of patients as secondary prevention (after ventricular arrythmias with hemodynamically instability).

**Figure 3:**
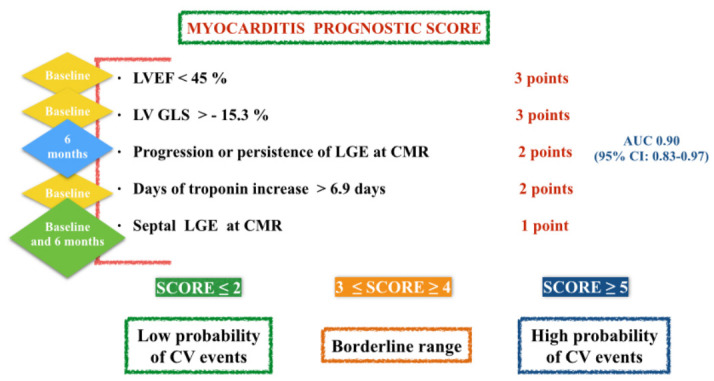
Myocarditis prognostic score.

We did not find significant differences regarding myocarditis recurrence and ventricular arrhythmias between the two groups (Table 3). Most deaths occurred in the first year after acute myocarditis and hospitalizations for HF that occurred in the first 3 years (Figure 3).

Patients with cardiovascular events had significantly lower values of LVEF at echocardiography (47 ± 15) compared to patients without events (56.19 ± 9.6, *p*-value 0.0006) and CMR (*p*-value 0.0018); lower GLS in absolute value (−17 ± 4 vs. −18.45 ± 2.78, *p*-value 0.04); and greater persistence of increased troponin over time (6 ± 2 vs. 4 ± 2, *p*-value < 0.0001).

Among clinical presentations, a significantly higher percentage of patients with events had dyspnea (*p*-value 0.0007); chest pain was significantly predominant in patients without events (*p*-value 0.03). A significantly higher percentage of patients with events had progression or persistence of LGE on 6 months CMR compared to patients without events (respectively 89% vs. 64%, *p*-value 0.01) and septal LGE on the baseline or 6 months CMR (19% of patients vs. 6%, *p*-value 0.048). Table 4.

Logistic regression analysis confirmed the significant association between cardiovascular events and lower values of LVEF at presentation (*p*-value 0.004), reduced GLS in absolute value at presentation (*p*-value 0.044), troponin increase for several days (*p*-value 0.014) at first hospital admission, septal LGE on any CMR (*p*-value 0.002) and persistence–progression of LGE on 6 months CMR (*p*-value 0.013). Table 5.

Using logistic regression and ROC analysis, we found that value of LVEF, GLS and longer time of persistently increased troponin were predictors of cardiovascular events, with the greatest sensitivity and specificity. Particularly, we found that a baseline LVEF value of <45% assessed by echocardiogram (AUC 0.65; 95% CI: 35 –55, *p*-value 0.02); a baseline GLS value more than −15.36 (AUC 0.67; 95% CI: −17.83 – −5.510, *p*-value 0.05); >6.9 days of persistence of increased troponin (AUC 0.81; 95% CI: 5 – 8, *p*-value 0.0001); the increase/persistence of LGE on 6 months CMR (AUC 0.69; *p*-value 0.02); and presence of septal LGE (AUC 0.65, *p*-value 0.04) were associated with a greater risk of cardiovascular events.

Multivariate logistic regression analysis confirmed that these parameters (LVEF, GLS, septal LGE, persistence of increased troponin and persistence-progression of LGE) were independent predictive factors for major cardiovascular events (*p*-value < 0.05).

In addition, we found that 10% of patients with complicated myocarditis had third-degree atrio-ventricular block. These patients had giant cell myocarditis as demonstrated in endomyocardial biopsy. No cases of cardiac sarcoidosis have been diagnosed and FDG-PET (2-Deoxy-2-[18F]fluoro-D-Glucose-positron-emission tomography) was performed in suspected cases.

Considering the laboratory test, we did not find significant differences in renal function values between patients with and without cardiovascular outcomes. We did not find significant differences in PCR values between patients with and without myocarditis.

### 3.3. Myocarditis Prognostic Score

Considering regression analysis results, we developed a multiparametric prognostic score based on these five items (LVEF, GLS, days of troponin increase, septal LGE, progression/persistence of LGE on 6 months CMR) attributing different points to each item based on the regression beta coefficient (Table 5). Therefore, 3 points were assigned to the presence of LVEF < 45% on echocardiogram; 3 points to a GLS value > −15.3%; 2 points to an increase/persistence of LGE on 6 months CMR; 2 points at troponin increase time > 6.9 days; and 1 point to the presence of septal LGE (Figure 3).

The score was found to be accurate in identifying patients at increased risk of cardiovascular events: AUC 0.90 (95% CI: 0.83–0.97), *p*-value < 0.0001. In particular, a score ≤ 2 had high sensitivity (97%; 95% CI: 90–99%) and low specificity (39%; 95% CI: 28–50%) in identifying cardiovascular events (AUC 0.68—95% CI: 0.59–0.77).

A score between 3 and 4 showed sensitivity of 100% but low specificity (8%) with AUC equal to 1. A score ≥ 5 showed sensitivity of 100% and specificity of 87% with AUC equal to 1, and *p* value < 0.0001; therefore, a score ≥ 5 seems had the greatest prognostic accuracy.

This score was calculated in the entire population and the association with major cardiovascular outcomes was assessed.

**Figure 4:**
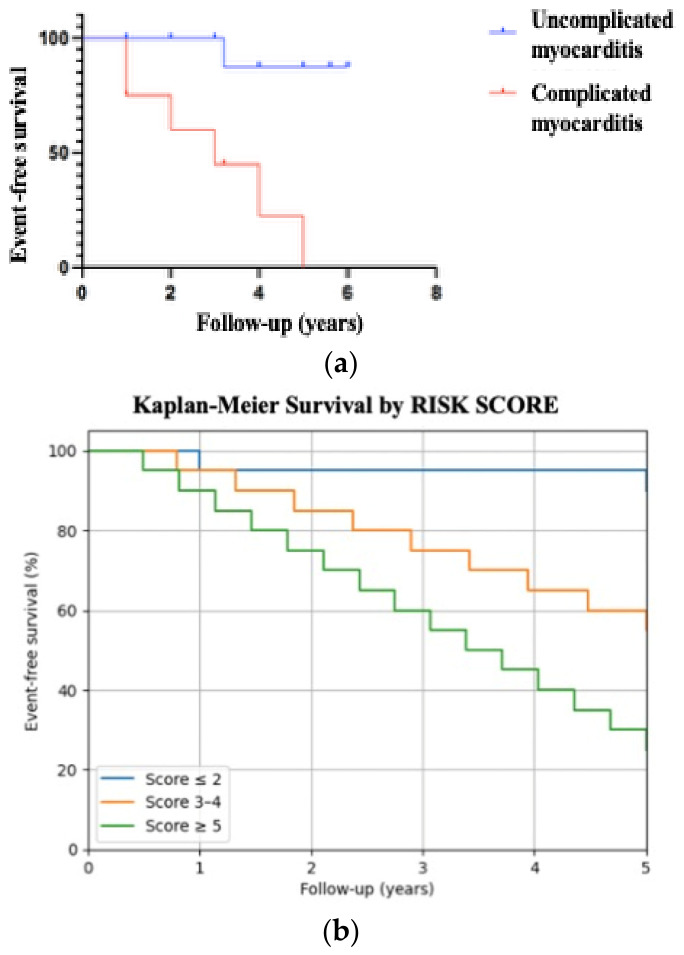
(**a**) Event-free survival Kaplan–Meier; (**b**) Kaplan–Meier survival by risk score.

We found a score ≤ 2 in 82% of patients without events compared to 7% of patients with events (*p*-value < 0.0001). A score between 3 and 4 was found in 35% of patients with events and 12% of patients without events (*p*-value 0.009). A score ≥ 5 was found in 58% of patients with events compared to 6% of patients without events (*p*-value < 0.0001; see Table 6, Figure 4a,b). Thus, a score ≤ 2 was associated with low probability of cardiovascular events; a score between 3 and 4 represents a borderline range; and a score ≥ 5 was associated with high probability of events.

## 4. Discussion

Our study showed a higher incidence of cardiovascular events (cardiovascular death, hospitalization for heart failure, ICD implantation, arrhythmias, heart transplant and HF development) in patients with complicated myocarditis compared to patients with uncomplicated myocarditis at presentation, using the myocarditis’s classification proposed by Ammirati [6,7].

These results are consistent with previous studies on myocarditis. In fact, in the Lombardy registry, Ammirati et al. showed that patients with complicated myocarditis at presentation were at higher risk of cardiovascular events (cardiac deaths and heart transplant) compared with uncomplicated cases that had a benign prognosis and low risk of subsequent left ventricular systolic dysfunction [7].

The classification in “complicated” and “uncomplicated” myocarditis seems to have an important prognostic role [7,8,9,10,11,12,13,14,15,16,17,18,19,20,21,22,23,24,25].

The novelty of our study was to propose a simple numerical and multiparametric prognostic score, including echocardiographic parameters, CMR data and cardiac biomarkers.

Particularly, our score included an important prognostic parameter such as LGE progression on 6 months CMR and it should be calculated at the first visit at 6 months to stratify the patient’s prognosis and to plan the most appropriate follow-up. The other existing studies evaluated single prognostic parameters in patients with myocarditis.

Aquaro et al. demonstrated that in patients with myocarditis and preserved LVEF, late gadolinium enhancement (LGE) in the mid-wall layer of the anteroseptal wall was associated with a worse prognosis regardless of LGE extension [26]. Another study showed that the presence of LGE without edema at 6 months CMR was associated with worse prognosis, particularly when distributed with a mid-wall septal pattern. Particularly, increased extent of LGE at 6 months CMR had a worse prognosis than decreased/unchanged LGE [27].

ECG can provide important prognostic information in patients with myocarditis.

For example, increase in QRS duration is an independent predictor of death and heart transplantation in patients with acute myocarditis [28].

Fragmented QRS is a known marker of fatal arrhythmias or cardiac adverse events in ischemic and non-ischemic cardiomyopathy patients. A study showed that fragmented QRS is an independent predictor of adverse events, particularly late-onset ventricular arrhythmias in patients with cardiac sarcoidosis [29].

Other studies showed that a wide QRS-T angle, low voltage and fragmented QRS were significantly associated with LGE in patients with myocarditis. A wide QRS-T angle, low voltage and prolonged QTc duration were associated with cardiovascular events in patients with myocarditis [30].

Echocardiographic parameters can have a prognostic role and they can help to identify a subclinical cardiac involvement in patients with myocarditis and preserved LVEF. For example, studies showed that in patients with acute myocarditis and preserved LVEF, not only GLS but also left atrial reservoir function, left atrial conduit function and left atrial stiffness index, as well as left atrial filling index, were impaired compared to healthy controls indicating ventricular diastolic dysfunction and elevated LV filling pressures [31,32].

Right ventricular function can also have a prognostic role. RV strain assessed by CMR is associated with cardiovascular events, heart failure hospitalization and death in patients with myocarditis [33]. Xu et al. proposed a risk prediction model for in-hospital mortality in patients with suspected myocarditis. They showed that a creatinine clearance rate < 60 mL/min, an age ≥ 50 years, ventricular tachycardia, a NYHA classification ≥ 3, male gender, and a troponin level ≥ 50 μg/L were the independent risk factors in patients with suspected myocarditis [34].

Baritussio et al. analyzed the predictive role of biventricular systolic function and LGE extent on CMR at diagnosis in patients with myocarditis [34].

In children with myocarditis, LVEF between 40 and 50% at admission and lactate dehydrogenase value have a significant prognostic value [35].

Different from other studies, in our study, we found that a lower LVEF at presentation (<45%), a lower GLS in absolute value (<15.3%), a persistent troponin increase (>6.9 days), the presence of septal LGE, and the progression/persistence of LGE on 6 months CMR are predictors of cardiovascular events in myocarditis patients. Troponin increase for several days predicted cardiovascular events according to literature data. In fact, Ammirati et al. suggested that an early increase with a rapid decline in troponin is generally associated with resolution or attenuation of the inflammatory process with a good prognosis; recurrent or persistently abnormal troponin levels may suggest relapse or persistent myocardial damage [6,7,8,9,10].

GLS assessed at echocardiography and CMR is also a predictor of events in several studies [36].

A multiparametric score including these items ≥ 5 had the greatest prognostic accuracy in our study. A score ≤ 2 was associated with low probability of cardiovascular events; a score between 3 and 4 represents a borderline range but it can identify patients at high risk of events. In our score, the duration of troponin elevation (6.9 days) had the best predictive value compared to the other parameters.

Thus, in addition to the other scores, our score is a practical tool to use not so much at the time of hospital admission, whose prognostic factors upon admission are widely known, but at the time of the first visit at 6 months when the evaluation of progression/regression of the LGE, in addition to the known prognostic factors at admission, can provide added value, especially in follow-up planning. Thus, myocarditis prognostic score might be integrated into clinical practice at the visit at 6 months, to guide therapeutic decisions and to personalize follow-up strategies.

For example, a low score at follow-up could help the clinician to decide to discontinue cardio-protective therapy or restart sports activity; a high score may suggest the clinician to continue therapy and do a close follow-up.

Considering myocarditis recurrences, a higher incidence of myocarditis recurrences was found in patients with uncomplicated myocarditis. It is known that several factors can promote relapse of myocarditis such as previous myocarditis and young age [37]. In fact in our study, patients with uncomplicated myocarditis were significantly younger than patients with complicated myocarditis and they could have a greater risk of exposure to the same trigger, during life. Also, genetic background can favor myocarditis recurrences and influence the prognosis [38,39,40].

Considering the arrhythmic risk, in our study, we did not find a significant difference between the two groups of patients with myocarditis. In fact, it is known that patients with myocarditis and preserved LVEF can also have risk of arrhythmias and other cardiovascular events.

For example, Gentile et al. showed that in patients with myocarditis “complicated” by ventricular arrhythmias at onset, the recurrence of arrhythmias during follow-up correlated with the extension of the LGE (≥2 segments) and the absence of edema at onset [41].

It is also known that patients with myocarditis and preserved LVEF and septal LGE at CMR and/or LGE increase on 6 months CMR had a worse prognosis than those with decreased/unchanged LGE [26,27]. Also, GLS at echocardiography or CMR can predict cardiovascular events in patients with myocarditis and preserved LVEF. These parameters included in the score (GLS and LVEF) have been assessed using 2D echocardiography and CMR. Particularly, LVEF was assessed using 2D echocardiography and CMR; GLS was assessed using 2D speckle tracking echocardiography.

CMR is the diagnostic gold standard for the assessment of LVEF. Unfortunately, LVEF and GLS have several limitations such as load dependency. In addition, 2D echocardiography has several significant disadvantages including poor image quality due to sonographer skill, poor acoustic windows, or other patient characteristics. The 2DE-LVEF is known to have large inter- and intra-observer variability [42]. Therefore, great attention must be paid to the evaluation of these parameters.

Thus, also considering the risk of cardiovascular events in patients with preserved LVEF and uncomplicated myocarditis, and considering the myocarditis’s classification proposed by Sinagra in low–intermediate and high risk [43], our study identified predictors of cardiovascular events in all patients with myocarditis.

Unfortunately, even if this score provides useful prognostic information, it is difficult to apply in some healthcare settings because it requires monitoring troponin levels over several days and a second CMR 6 months after hospital admission.

## 5. Conclusions

Our study confirms the higher incidence of cardiovascular events in complicated myocarditis and the need for classification into “complicated” and “uncomplicated” myocarditis, but it highlights the importance of other parameters that can add prognostic information in all patients with myocarditis. In fact, classification in “complicated” and “uncomplicated” myocarditis could provide important prognostic information at hospital admission. In patients with myocarditis, our risk score could help to evaluate the prognosis and to plan an adequate follow-up, especially at visit at 6 months. Unfortunately, this score was not validated externally and the results are applicable only in the study cohort.

The “myocarditis prognostic score” aims to be a practical tool for the clinician to use to remember the main prognostic factors in myocarditis, stratify the risk of cardiovascular events and plan the most appropriate follow-up in myocarditis patients. This score should be used during the first post-discharge clinical check-up at 6 months, after repeating CMR.

Surely other studies are needed to validate and consolidate this score.

## 6. Study Limitations

Our study has several limitations. The first being a small number of patients and the short duration of the follow-up; therefore, larger and multicenter studies are warranted to confirm our findings and a longer follow-up.

Myocarditis prognostic score lacks external validation. It needs to be validated in a larger population: the absence of score validation in at least another independent population is a major limitation of the study. Other limitations of the study are: the absence of prognostic markers such as arrhythmic burden and genetic predisposition; the absence of extensive data on medical therapy; the absence of “electrical” parameters (for example, ventricular extra beats, QRS abnormalities such as fragmentation, inducible arrhythmias). Genetic assessment has not been performed in patients with cardiovascular events and it will be performed in the future in a multicenter study. In fact, the main focus of the study was to identify a score that included cardiovascular imaging parameters.

In the future, electrical parameters already evaluated in other diseases could be included in a risk score, in patients with myocarditis. Other limitations of the study are the load dependence of the echocardiographic parameters included in the score (LVEF and LV GLS) and the use of 2D echocardiography due to the unavailability of 3D probe and software.

In addition, the variation of LV-GLS parameters between the various vendors is another limitation of the study. In this study, the value of LV-GLS was assessed using GE vendor. Another limitation of the study was the failure to evaluate the extracellular volume and T1 mapping in the CMR.

## Figures and Tables

**Table 1:** Clinical characteristics of patients with myocarditis at hospital admission.

	General Population (98 pts)	Complicated Myocarditis (31 pts)	Uncomplicated Myocarditis (67 pts)	*p*-Value
**Demographics data**				
Age, years	27 (20–40) 30.75 ± 13.74	34 (24.5–49.5) 38.95 ± 18.33	25 (19–36) 29.4 ± 12	0.0027
Gender, female, n pts (%)	26 (26%)	9 (28%)	17 (25%)	0.8
Body mass index (kg/mq)	25 ± 4.65	26.4 ± 3.36	25 ± 4	0.7
Arterial hypertension, n pts (%)	10 (10%)	2 (6%)	8 (11%)	0.4
Smoking, n pts (%)	30 (31%)	8 (25%)	22 (32%)	0.48
Dyslipidemia, n pts (%)	7 (7%)	2 (6%)	5 (8%)	0.7
Diabetes mellitus, n pts (%)	7 (7%)	4 (12%)	3 (5%)	0.21
Beta-blockers, n pts (%)	35 (36%)	13 (43%)	22 (34%)	0.39
ACE-I/ARB, n pts (%)	41 (41%)	16 (53%)	25 (38%)	0.16
Anti-inflammatory therapy, n pts (%)	48 (49%)	12 (40%)	36 (54%)	0.19
**Clinical data**				
Dyspnea, n pts (%)	17 (17%)	15 (50%)	2 (3%)	<0.0001
Chest pain, n pts (%)	83 (84%)	21 (68%)	62 (92%)	0.0024
Syncope, n pts (%)	3 (3%)	2 (9%)	1 (1%)	0.04
Fever within 30 days before, n pts (%)	37 (39%)	18 (58%)	19 (28%)	0.0045
Previous myocarditis, n pts (%)	6 (6%)	3 (10%)	3 (5%)	0.35
Fulminant myocarditis, n pts (%)	7 (7%)	7 (22%)	0 (0%)	0.0001
**Laboratory test**				
CRP (mg/L)	46 ± 72 20 (11–47)	64 ± 75 27 (15–120)	38 ± 64 20 (10–42)	0.07
Troponin (ng/L)	1283 ± 1940 632 (253–1421)	1450 ± 2167 718 (341–1397)	1253 ± 1922 652 (281–1421)	0.65
NT-proBNP (mg/L)	995 ± 1698 430 (173–891)	1414 ± 1498 662 (351–2160)	827 ± 1767 317 (106–699)	0.11
Time of increased in troponin (days)	5 ± 3 3 (2.5–8)	7 ± 3 8 (6–8)	4 ± 2 4 (2.5–5.5)	<0.0001

ACE-I: Angiotensin-converting enzyme inhibitors; ARB: angiotensin receptor blockers; CRP: C-reactive protein; NT-proBNP: N-terminal prohormone of brain natriuretic peptide. Pts: patients.

**Table 2:** Echocardiographic parameters, CMR data and other instrumental parameters in patients with myocarditis during hospitalization.

	General Population (98 pts)	Complicated Myocarditis (31 pts)	Uncomplicated Myocarditis (67 pts)	*p*-Value
Coronary angiography, n pts (%)	34 (34%)	16 (53%)	18 (27%)	0.01
Coronary tomography, n pts (%)	20 (20%)	15 (48%)	5 (7%)	<0.0001
Endomyocardial biopsy, n pts (%)	20 (20%)	20 (64%)	0 (0%)	<0.0001
**Electrocardiogram**				
Normal, n pts (%)	27 (26%)	5 (15%)	22 (33%)	0.06
ST elevation, n pts (%)	46 (47%)	12 (37%)	34 (50%)	0.23
Third degree atrio-ventricular block, n pts (%)	3 (3%)	3 (10%)	0 (0%)	0.0089
**Echocardiography**				
LVEF (%)	60 (52.5–60) 55 ± 11	38 ± 12 42 (30–49)	60 ± 4 60 (58–60)	<0.0001
EDV (mL)	100 ± 28 98.5 (81–114)	118 ± 39.2 108 (95–129)	95 ± 41 95 (80–113)	0.01
GLS (%)	−18 ± 3 −14.5 (−18; −30)	−14 ± 3 −15 (−17; −11)	−19 ± 2 −19 (−21; −17)	<0.0001
Pericardial effusion, n (%)	17 (17%)	6 (21%)	11 (17%)	0.6
**Cardiac magnetic resonance**				
LVEF (%)	58.7 ± 9.4 59 (54–64)	39 ± 10 43 (36–52)	61 ± 8 61 (55–66)	<0.0001
EDV (mL)	145 ± 40 139 (119–167)	175 ± 58 158 (133–209)	140 ± 33.6 137 (114–167)	0.0003
Edema, n pts (%)	98	31 (100%)	67 (100%)	
Delayed enhancement, n° pts (%)	83 (85%)	29 (96%)	54 (81%)	0.04

EDV: end-diastolic volume, GLS: global longitudinal strain, LVEF: left ventricular ejection fraction. pts: patients.

**Table 3:** Cardiovascular events in all populations with myocarditis.

	General Population (98 pz)	Complicated Myocarditis (31 pts)	Uncomplicated Myocarditis (67 pts)	*p*-Value
Death, n° pts (%)	7 (7%)	6 (19%) cardiovascular death	1 (1%) other cause of death	0.0010
Hospitalization for heart failure, n° pts (%)	9 (9%)	9 (29%)	0 (0%)	0.01
Heart transplant, n° pts (%)	5 (5%)	5 (16%)	0 (0%)	0.0008
Myocarditis recurrence, n° pts (%)	16 (16%)	4 (12%)	12 (18%)	0.45
Ventricular arrhythmias, n° pts (%)	3 (3%)	2 (6%)	1(1%)	0.14
ICD implantation, n° pts (%)	10 (10%)	9 (29%)	1 (1%)	<0.0001
Heart failure development	15 (15%)	15 (48%)	0 (0%)	<0.0001

**Table 4:** Main differences between patients with and without cardiovascular events.

	Cardiovascular Events (26 pts)	No cardiovascular Events (72 pts)	*p*-Value
Gender, male	17 (65%)	69 (83%)	0.05
Age, years	35 ± 20	29.8 ± 12	0.10
Body mass index	23.7 ± 3.8	25.29 ± 3.68	0.059
Arterial hypertension, n° pts (%)	4 (15%)	6 (9%)	0.38
Smoking, n° pts (%)	7 (26%)	23 (32%)	0.57
Dyslipidemia, n° pts (%)	2 (7%)	5 (8%)	0.87
Fever, n° pts (%)	9 (34%)	28 (39%)	0.65
Chest pain, n° pts (%)	19 (73%)	64 (90%)	0.03
Dyspnea, n° pts (%)	10 (38%)	7 (9%)	0.0007
Syncope, n° pts (%)	2 (7%)	1 (2%)	0.22
Troponin T hs (ng/L)	1935 + 2713	1505 ± 2405	0.44
CRP (mg/L)	50 ± 62	47.5 ± 79	0.88
NT-pro BNP (mg/L)	1361 ± 1256	905 ± 1213	0.1
ST elevation, n° pts (%)	12 (46%)	39 (47%)	0.9
LVEF Echo (%)	47 ± 15	56.19 ± 9.6	0.0006
EDV (mL)	101 ± 23	102 ± 29	0.87
GLS (%)	−17 ± 4	−18.45 ± 2.78	0.04
LVEF RM (%)	52 ± 15	59.91 ± 8.81	0.0018
N° of segments with edema	2 ± 2	3.18 ± 3	0.06
N° of segments with LGE	5 ± 4	3.8 ± 2.9	0.1
Days of increase in Troponin	6 ± 2	4 ± 2	<0.0001
Reduction in LGE, n° pts (%)	3 (11%)	25 (36%)	0.01
Septal LGE, n° pts (%)	5 (19%)	5 (6%)	0.048

EDV: end-diastolic volume, GLS: global longitudinal strain, LGE: late gadolinium enhancement, LVEF: left ventricular ejection fraction, NT-proBNP: N-terminal prohormone of brain natriuretic peptide, CRP: C-reactive protein. pts: patients.

**Table 5:** Logistic regression in patients with cardiovascular events.

Prognostic Factors	Hazard Ratio [95% CI]	*p*-Value	Regression B Coefficient	Points in the Score
LVEF echo (%)	4.765 (0.781–32.47)	0.004	3.561	3
GLS (%)	27.21 (0.55–32)	0.044	3.304	3
Days of increase in troponin	1.99 (1.42–3)	0.014	2.893	2
Septal LGE at CMR	0.15 (0.07–0.275)	0.002	1.879	1
Progression or persistence of LGE at 6 months CMR	0.07 (0.016–0.18)	0.013	2.708	2

**GLS**: global longitudinal strain, **LGE:** late gadolinium enhancement, **LVEF**: left ventricular ejection fraction.

**Table 6:** Differences between patients with and without cardiovascular events for each score category.

	Myocarditis Prognostic SCORE ≤ 2, n° pts (%)	Myocarditis Prognostic SCORE 3–4, n° pts (%)	Myocarditis Prognostic SCORE ≥ 5, n° pts (%)
Cardiovascular events (n. pts 26)	2 (7%)	9 (35%)	15 (58%)
No cardiovascular events (n. pts 72)	59 (82%)	9 (12%)	4 (6%)
*p*-value	<0.0001	0.009	<0.0001

## Data Availability

The original contributions presented in this study are included in the article. Further inquiries can be directed to the corresponding author.
